# Intergenerational Virtual Program: Promoting Meaningful Connections Across the Lifespan During the COVID-19 Pandemic

**DOI:** 10.3389/fpubh.2021.768778

**Published:** 2021-12-20

**Authors:** Ann Kennedy-Behr, Edoardo Rosso, Sarah McMullen-Roach, Angela Berndt, Ashleigh Hauschild, Hannah Bakewell, Kobie Boshoff, Daniel Antonello, Badakhsh Jeizan, Carolyn M. Murray

**Affiliations:** ^1^Occupational Therapy, UniSA Allied Health & Human Performance, University of South Australia, Adelaide, SA, Australia; ^2^ACH Group, Mile End, SA, Australia; ^3^International Centre for Allied Health Evidence, University of South Australia, Adelaide, SA, Australia; ^4^Child Care Network and Creche Services, TAFE SA, Adelaide, SA, Australia

**Keywords:** active aging, intergenerational programs, children, older adult, technology, health and well-being

## Abstract

Intergenerational programs have long been identified as a way of promoting health and well-being for participants. Continuing such programs during pandemic restrictions is challenging and requires a novel approach. This community case study describes the use of co-design to create a high-level intergenerational program model, adapt it to specific community needs, and deliver it virtually with the aid of modern communication technology. Interviews conducted after the program had finished indicated that despite the challenges and limitations of the virtual environment, meaningful connections were achieved across three generations. The high-level program model may serve as a basis for other programs wanting to explore this area.

## Introduction

The existence and benefits of non-familial intergenerational programs for children and older adults are well-established (1–4). Emerging as a response to changing sociodemographic trends in the 1960's in the US, the scope and heterogeneity of inter-generational programs have proliferated (5, 6). Typically, intergenerational programs are defined as services that aim to increase sharing, interaction, or exchange between two generations, particularly when separated due to changes in social structures. These initiatives enable the strengths of each age group to enhance the experience or quality of life of the other (7). More simply, intergenerational programs include “activities that bring old and young together for their mutual benefit” (8). For older people there is evidence of self-reported improvements in health and depressive mood, self-esteem and confidence, enjoyment, satisfaction and happiness, improved interactions and relationships with others (1, 2, 6).

Reported benefits for children include supporting them to develop a positive attitude to and more knowledge of aging (6, 9), skill development and character building, mood and enjoyment, feeling helpful and having a friend (4).

Quality outcomes in intergenerational programs are achieved when implementation is theoretically sound and evidence based, and the high level of logistical, staffing and curricular work or training is addressed (6, 10). Evidence suggests the absence of these characteristics, with a concurrent assumption that “simply bringing younger and older people together will result in meaningful relationships” (11), is a “fatal flaw” of some intergenerational programs resulting in them being only short-lived (10).

One form of service delivery involves the co-location of pre-school children and vulnerable seniors in fully integrated shared site intergenerational programs (SSIPs) to meet the diverse needs of families without duplication of services (10). The SSIP is a way to link young and old, eliminate transport issues and costs, schedule shared activity, create opportunity for informal activity and ensure sustainability (10). However, such day services for young and old are not yet a mainstream option in Australia, and as such most intergenerational programming involves the two cohorts meeting for shorter timeframes. When one of the participant groups includes people with dementia or frailty, it is more likely the child participants will travel to the older people. However, with the advent of the COVID-19 pandemic and the immediate physical distancing requirements, the option for face-to-face interaction was halted.

Using technology as a mode for delivering health programs is also well-established with telehealth being in existence for almost 60 years (12). However, the gaps in services and supports identified and the need to innovate during the COVID-19 pandemic accelerated the use of telehealth interventions in new ways with novel populations (13). A recent scoping review of 77 studies exploring technologies for fostering intergenerational connectivity and relationships found this is an early and emergent field, e.g., over half the studies were reported in conference proceedings (14). Only two studies were conducted in assisted living facilities and only one used technology, being an Apple iPad® mediated art class (15); no studies reported participants located in different environments or technology being used to connect participants for inter-generational programs activity.

This community case study reports on a pilot project using virtual conferencing to deliver a structured, co-designed intergenerational program with children located in an inner-city childcare center and residents of a suburban aged care facility.

## Context

The Australian Government's Aging in Place policy has changed the way services are provided to its older citizens. Of the 1.3 million consumers of aged care services in 2019–2020, only one third were living in residential care (16) with the majority still living in their own homes. A corollary of this is that those who are in residential facilities tend to be high need and/or frail. The most recent figures indicated that 58% of residents were over 85 years of age (16) and 51.9% had a diagnosis of dementia.

The intergenerational program, which is the focus of this report, took place in Adelaide, South Australia, a city of 1.3 million, in an inner-city childcare center and a suburban aged care facility located 10 km away. This project fits in the context of South Australia's Plan For Aging Well 2020–25 (17), which identifies meaningful connections as a strategic priority to support older people to age well, both in the community and in residential facilities, and it proposes that challenging ageism is an important condition to achieve that.

The intergenerational program was the product of co-design between the aged care provider, university staff and students, childcare staff and aged care residents and had six phases: scoping, planning, co-design, pilot, evaluation, and reporting.

Co-design, also referred to as participatory design (18, 19), is relatively new in healthcare and focuses on empowering the participants most affected by the design (19), in this case, the residents and children. Participants are co-designers, involved at all stages of the design process rather than being passive recipients of the design or program. Co-design is suitable to be used with vulnerable participants. By necessity, co-design involves power-sharing, and giving a voice in decision-making to those who are often left unheard (18).

Older adults, living in residential care, and young children are both vulnerable groups. Bringing two vulnerable groups physically together can be logistically complex and was made more difficult by the COVID-19 pandemic. The safety of both groups needed to be ensured and appropriate compliance checks needed to be in place. Although the intergenerational program was originally planned for delivery face-to-face, COVID-19 restrictions throughout 2020 and much of 2021 excluded young children from visiting aged care facilities and so a virtual program was designed instead.

**Figure 1:**
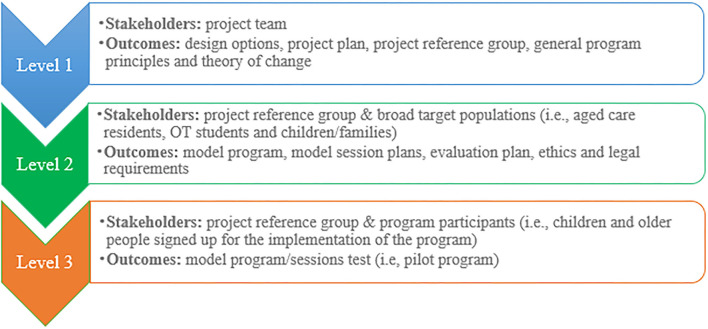
The process of co-design.

The co-design phase was conducted between August and December 2020 and aimed to involve all stakeholders in the development of a high-level program model. It was conceived as a three-level exercise starting from broad principles and concluding with the test of actual locally responsive intergenerational activities designed in partnership with participants. Figure 1 summarizes the co-design approach.

In this phase, aged care residents, staff, children and their families and occupational therapy students were consulted in depth regarding their interest in an intergenerational program, their hopes and any concerns that they might have. These were incorporated into the original design with the key objectives of increasing social connectedness for both older people and young children. The project also aimed to explore and develop new opportunities for older people to actively shape services that support aging well.

**Figure 2:**
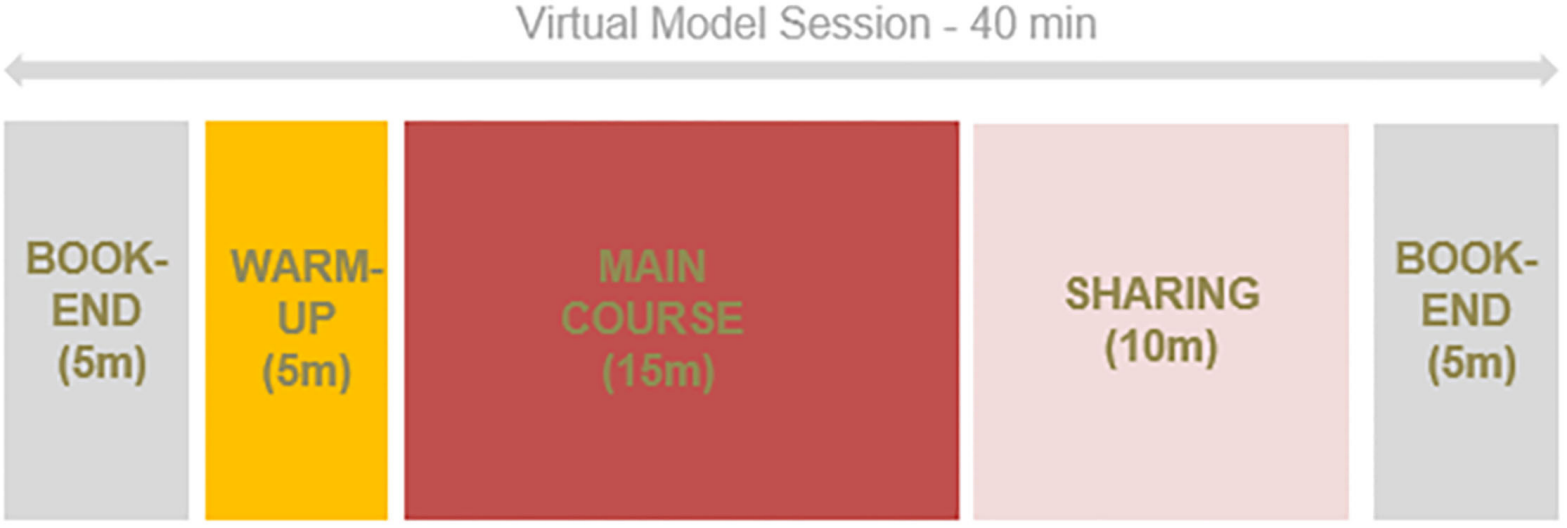
High-level model of a virtual intergenerational session.

Following this, the key stakeholders developed a broad program model that was feasible within the restrictions but also contained flexibility and ensured the needs of both children and aged care residents were represented. The basic structure of the model consisted of re-occurring “book-ends” to start and finish each session, a warm-up and a main activity. Staff with experience working in aged care and childcare proposed basic themes such as gardening, cooking, travel, going to the beach, which served as a starting point for customization with the participants (see Figure 2).

At the beginning of the pilot phase, two occupational therapy students took the basic structure and brought the ideas of the program to residents. The students spent time with the residents building relationships and explaining what would be involved. Of the fifteen people identified as possible participants, ten chose to participate and either they or their next of kin signed consent forms. Possible participants were identified by the aged care facility's lifestyle coordinator. Residents with behaviors of concern were excluded from the program. As this was a health promotion project, diagnoses were not recorded nor relevant. The common reason for participation was a desire to interact with children as many residents had grandchildren they did not see often. Residents also participated because they wanted to engage with other like-minded residents. Reasons given for parents enrolling their children in the program included their child not having grandparents and wanting to develop their child's connection to the wider community.

The two occupational therapy students, in partnership with staff of all three organizations involved, spent 3 weeks adapting the broad model program to the specific needs and interests of those who enrolled in the pilot. This involved fine-tuning all the proposed activities and overcoming logistic and coordination problems, including how to best introduce participants to the technology. This included repeated interactions with the aged care residents *via* informal conversation and *via* play with the children. The student facilitators spent one morning a week interacting with the children, and one morning a week interacting with the residents. The interactions allowed the facilitators to develop a relationship with the children and residents, and to learn about them as individuals. Further, the interactions guided the themes and activities of the weekly sessions, as they were based on the information gained from discussions and feedback from the participants. Ultimately, the interactions ensured the weekly sessions were client-centered, and based on tailored activities, interests, and themes. Following the program design, the pilot intergenerational program was run as described below.

### Detail to Understand Key Programmatic Elements

The final program consisted of a 45-min session held once a week for 5 weeks. Children participated *via* Zoom Cloud Meetings from their childcare center, supported by their teachers and one occupational therapy student. The aged care residents met in the facility's clubrooms which had a large drop-down screen for the projection of the Zoom connection. They were supported each week by an occupational therapy student and university supervisor, the aged care facility lifestyle coordinator and a volunteer.

Older adult participants ranged in age from 77– 94 years and the children were aged between 3–5 years. Each session followed the same pattern, starting with a welcome song (“Wheels on the bus”), themed activities, a refreshment, time to chat and then a goodbye song (“You are my sunshine”). An additional component was added to the broad model developed in the planning phase. Due to concerns regarding establishing relationships and connections being minimized on a virtual platform, a sharing component was added to the plan to assist with individual interactions, knowledge sharing and relationship development.

In addition to the weekly Zoom session between the residents and children, an extra session was run with just the children a day prior to the intergenerational program. These sessions supported the children's ability to participate in the virtual sessions and were shorter but had the same theme with a different activity. The additional session allowed time to familiarize the children with the Zoom sessions and assisted with developing relationships with the children. The children sang the same “book-ends” and the facilitators discussed the weekly theme with the children and their “Grandfriends” (20). ^1^ The children-only sessions were also an opportunity for the children to create something for their Grandfriend, which could be given to them the next day and discussed in the session. This mimicked a relationship between a grandchild and their grandparent, wherein the grandchild would make something for their grandparent and give it to them. Examples of things made included musical instruments, painted a pot plant, “baked” cupcakes, and made an Easter card for their Grandfriends.

Time was spent interacting and establishing relationships with the participants to guide the themes used within the pilot. Interactive activities were chosen based on participants' interests, while ensuring the activities enabled inclusion of everyone involved and appropriateness for both generations. Themes included “Getting to Know You, Rhythm, Gardening, Farms, and Easter,” as participants shared an interest in music and gardening, some residents had lived on farms and the children enjoyed farm animals, and Easter brought about mutual excitement among the participants. The participants' culture was considered in session planning, and the sessions provided them with the opportunity to share their culture (particularly within the Easter session), which supported knowledge sharing, learning and connection. At the conclusion of the pilot program, interviews were conducted with adult participants to involve them in the evaluation of the project and to gain an in-depth understanding of their perspectives. Six residents agreed to be interviewed and two staff members (see Table 1). Interviews were chosen to allow participants time to speak. All participants were familiar with the interviewer (an occupational therapist) and interviews gave participants the opportunity to express their opinions. The interviews were audio-recorded, transcribed and then analyzed using independent line by line coding (21) by two authors. These were then discussed and further developed into themes which are presented and discussed below.

**Table 1:** Participants' demographics.

**Name (pseudonym)**	**Age**	**Sex**	**Resident or staff member**
Ruby	88	F	Resident
Eileen	88	F	Resident
John	90	M	Resident
Peggy	84	F	Resident
Vera	92	F	Resident
Bob	86	M	Resident
Rosa	77	F	Resident
Beryl	94	F	Resident
Flynn	–	M	Staff — 10 years' experience
Roger	–	M	Staff — 17 years' experience

## Results

Five key themes were identified from the interviews with participants and staff. These themes included: “connection, skilled facilitators, exploration of past and present roles, a wish for continuity, and online challenges.”

Findings are evidenced by quotes in text, pseudonyms are used to differentiate between participants, and different groups are identified by abbreviations R (resident) and S (Staff).

### Connection

The residents spoke of the connection they were able to develop with the children, even though the connection was developed and maintained over a virtual platform.

This connection was fostered through the use of shared occupation and experiences — which was reflected in the interview with Vera (R) “*I think the painting and that sort of thing was the best part… they were participating, and we were participating”*. This was also reflected through Flynn's statement “*there is a purpose and activity and that's what they come for”* (S).

When discussing highlights of the group three of the five residents spoke of the shared occupations where the children completed part of the task (i.e., painting the pot) and the resident finished the task (i.e., planting a plant). The children also made cards for the residents for Easter which were mentioned in the interviews “*I shall treasure this because I can't believe the note*” (Ruby, R). In this statement Ruby was referring to a touching note from a child that expressed feelings of care and love toward her.

The connection formed between the participants was emphasized by Eileen (R), “*they gelled with us…they wanted to see us again…they wrote notes to us*”. The residents also reflected on the level of connection with the younger people and the “genuine” nature of this connection. This sentiment was reflected by the children who embraced the title of “Grandfriends.” The use of this terminology demonstrated a “language of connecting with older people” [Roger(S)].

The sense of connection was strengthened through the self-identified grouping of the younger and older generations to the exclusion of the facilitators' (who were not part of either generation). Based on the interviews it appeared there was an “us and them” mentality with the older adults and children being “us” and the facilitators being “them”. This exclusive clique was reflected through the interviews; “*contact between [old and young] is very important in all walks of life, from when you start walking*” — Peggy (R), and “*I think everybody was really impressed, old and young*” Eileen (R). In fact, one of the older participants referred to the “adults” (facilitators) as being separate from the main group. This observation was supported through a staff member identifying that the “third generation [the student facilitators]” were important in bridging the age gap.

### Skilled Facilitators

A key theme that was identified was the skill of the facilitators in running the group and managing the technology, group dynamics and the children's engagement.

The areas that were commended were the facilitators' use of shared activities, adapting to an online environment and supporting the children to stay engaged.

In particular, their ability to adapt to technology was mentioned by participants and staff, “*I did not think we'd be able to do a group with another group at the other end, but they made it work really well*” [Flynn (S)] and “*whoever you are, to generate energy through a screen, to connect people, it is almost impossible to do*” [Roger (S)].

The residents all identified being surprised and impressed by the children's levels of engagement and the role of the facilitators in enabling this, “*the control she had over those children for their ages… it was amazing*” (Ruby, R.) and “*She had the class right under her [control]*” (Eileen, R).

In terms of the program itself, the residents identified that the group had run well, and this was confirmed with examples of activities completed. One participant, Vera stated “*I thought it was very good the way it was done*” (R.), this was supported by Ruby (R.) who identified that despite the need to run the group online “*everything else flowed so well*.”

### Exploration of Past and Future Roles

Each of the residents described their interaction with the group and the children through the lens of their own experience. This often led to an unprompted exploration of roles they had previously held in their families and/or the workplace. These roles were celebrated with a sense of achievement and pride.

For example, one participant reminisced on her previous experiences as a teacher and saw a relationship between this and her ability to easily engage with the children: “*It may have been that I have had a bit of training in that area*” (Ruby, R). Another spoke of her role as a mother “*I've been with children a lot, with six of our own*” (Eileen, R) and one shared his experience as a grandfather raising his grandson. Those who had less experience with children spoke of their upbringing and exposure to older adults when they themselves were a child “*I had a lot of contact with older people and that was, I don't know, just part of life*” (Peggy, R).

Participants were also able to be future focused, thinking about the children's future and the future of technology, and the modern world. Bob (R) shared a sense of wonderment at the children's future; “*I wonder what memories the kids will have*.” This finding portrayed the theme of hope for the future and a desire to be engaged in that future “*That's why I want to stick around for a few years, because I want to see what's going to happen*” (Ruby, R).

### A Wish for Continuity

All participants and staff expressed their enjoyment in the program and a wish for the program to continue, but this theme was most strongly shared by all the residents. “*I think it should keep going*” (Peggy, R.), “*I did enjoy it very much actually*” (Vera, R.) “*I was sorry when it finished*” (Eileen, R.).

Some of the residents reported in the interviews that the group was still being raised for discussion in their communal dining room. Many residents also reported that the timeframe (5 weeks) went very quickly, and they were “saddened” when the program was due to end.

### Online Challenges

Despite the positive outcomes and enjoyments of participants — both staff and residents expressed a desire for the program to run face to face — whilst acknowledging the merits of technology to enable it to happen during COVID-19 limitations.

In particular the staff reported disappointment at the program needing to move online “*I think we had our reservations about it being virtual*”- Flynn (S) and their hopes that it could have been run in person “*We were hoping for these sessions to be face to face*” – Roger (S).

Although the staff identified challenges with being online — the residents reported that the program ran well-despite the modifications, “*It is a shame they couldn't come here- but never mind*” (Vera, R). Residents were also surprised at the children's adaptability to the use of technology. When speaking about the use of technology, one resident discussed the children's ability to adapt to it “*[that was] the way that it worked, and they accepted it*” (John, R.).

## Discussion

This project tested an intergenerational program conducted virtually. Initially the project was anticipated to run face to face — however changes in COVID-19 restrictions forced the group into an online environment. To the best of our knowledge, this is the first study to report on the use of technology and occupation to facilitate the development of these connections.

Despite concerns that this shift would limit engagement and connection, the project was a success and this outcome opens up the potential for more diverse and innovative approaches to intergenerational connection.

Based on the interviews with the participants, it was evident that a bond was able to be formed between the children and older adults over the course of the project. The children readily adopted the term “grandfriends” indicating a special relationship built on pillars of friendship rather than service delivery, obligation, or family ties. The importance of friendship for older people in terms of physical, social and mental well-being is well known (22) and there has been growing investigation into the importance of “befriending”(23, 24). Befriending refers to a relationship where a younger person becomes friends with an older person and interactions are based around topics that are meaningful to the older person (23). In previous studies, befriending is implemented by volunteers and the benefits to the older adult receiving the “befriending” are captured. However, there has been little focus on the concept of reciprocity and what benefit the older adult brings to the relationship (25).

Analysis of the interviews suggested that this bond was enabled through the use of shared occupations and enhanced through a shared commonality: vulnerability.

The use of meaningful occupation to enable engagement, participation and well-being forms the foundation of occupational therapy (26). Sharing occupations is known to support the development of connection between groups and individuals and has long been used as a therapeutic tool (27–29). This was reflected within the program where participants reminisced about the activities and how they were able to share them together, albeit online.

## Conclusion

Intergenerational programs can facilitate connection even when conducted virtually. This innovative program demonstrated that while face to face is preferred, virtual interaction can still support a sense of belonging across generations. There was a unanimous response from residents and staff that the program should continue. It created meaning and connection and was a source of joy. More resources may be needed to deal with the technological challenges or ongoing technical advancements and the quality of the programming relies on careful attention to co-design and evaluation.

## Acknowledgment of Any Conceptual or Methodological Constraints

This was a pilot program conducted over 5 weeks with two vulnerable populations, aged care residents and very young children. This placed constraints on what outcome measures could be used and who could be interviewed. Not all residents were able to be interviewed due to limited verbal capacity. The findings of this project cannot be generalized to all aged care facilities however, they do provide a starting point and initial evidence on which longer and more in-depth projects can be based.

## Data Availability Statement

The raw data supporting the conclusions of this article will be made available by the authors, without unduereservation.

## Ethics Statement

The studies involving human participants were reviewed and approved by University of South Australia's Human Research Ethics Committee. Written informed consent to participate in this study was provided by the participants' legal guardian/next of kin.

## Author Contributions

AK-B: led the ethics application, supervised delivery of program, conducted all interviews, and co-wrote the manuscript. ER: led the Child Care in Aged Care project and contributed to the manuscript. SM-R: contributed to the development of the research protocol, data analysis, and manuscript. AB: contributed conceptually to the project and contributed the manuscript. AH and HB: facilitated the program and contributed to the manuscript. KB: contributed to the development of the research protocol and data analysis. DA and BJ: contributed conceptually to the co-design and delivery of the program. CM: contributed the development of the research protocol, the co-design process, data analysis, and manuscript. All authors contributed to the article and approved the submitted version.

## Funding

The Child Care in Aged Care project was supported by Office for Aging Well, SA Health (Government of South Australia), through a Strategic Projects Grant. The University of South Australia provided in-kind support.

## Conflict of Interest

ER and DA are employed by ACH Group. The remaining authors declare that the research was conducted in the absence of any commercial or financial relationships that could be construed as a potential conflict of interest.

## Publisher's Note

All claims expressed in this article are solely those of the authors and do not necessarily represent those of their affiliated organizations, or those of the publisher, the editors and the reviewers. Any product that may be evaluated in this article, or claim that may be made by its manufacturer, is not guaranteed or endorsed by the publisher.

## Footnotes

^1^“Grandfriends” was chosen as the term to describe the relationship between the children and residents as it reflects the intergenerational component but does not assume kinship. This term was adopted from Low et al. (19).
